# Retinal Stem Cell ‘Retirement Plans’: Growth, Regulation and Species Adaptations in the Retinal Ciliary Marginal Zone

**DOI:** 10.3390/ijms22126528

**Published:** 2021-06-18

**Authors:** Amanda Miles, Vincent Tropepe

**Affiliations:** Department of Cell & Systems Biology, University of Toronto, 25 Harbord Street, Toronto, ON M5S 3G5, Canada; amanda.miles@mail.utoronto.ca

**Keywords:** retina, neurogenesis, development, myopia, hyperopia, stem cell, vertebrate

## Abstract

The vertebrate retina develops from a specified group of precursor cells that adopt distinct identities and generate lineages of either the neural retina, retinal pigmented epithelium, or ciliary body. In some species, including teleost fish and amphibians, proliferative cells with stem-cell-like properties capable of continuously supplying new retinal cells post-embryonically have been characterized and extensively studied. This region, termed the ciliary or circumferential marginal zone (CMZ), possibly represents a conserved retinal stem cell niche. In this review, we highlight the research characterizing similar CMZ-like regions, or stem-like cells located at the peripheral margin, across multiple different species. We discuss the proliferative parameters, multipotency and growth mechanisms of these cells to understand how they behave in vivo and how different molecular factors and signalling networks converge at the CMZ niche to regulate their activity. The evidence suggests that the mature retina may have a conserved propensity for homeostatic growth and plasticity and that dysfunction in the regulation of CMZ activity may partially account for dystrophic eye growth diseases such as myopia and hyperopia. A better understanding of the properties of CMZ cells will enable important insight into how an endogenous generative tissue compartment can adapt to altered retinal physiology and potentially even restore vision loss caused by retinal degenerative conditions.

## 1. Introduction

**Figure 1:**
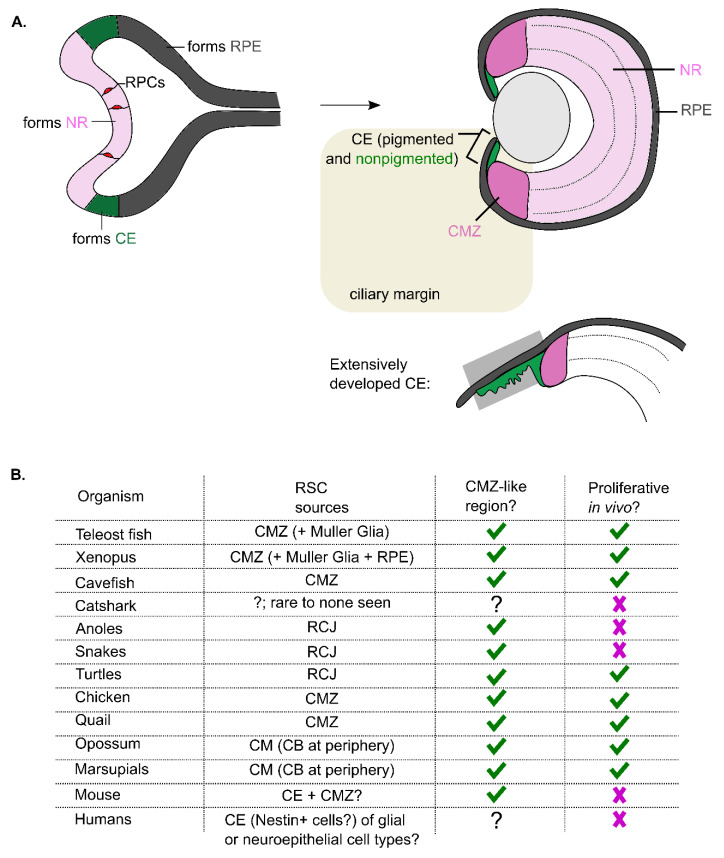
Conservation of a CMZ-like region across different species. (**A**) Schematic of the development and specification of cells in the eye into the retinal pigmented epithelium (RPE, *grey*), neural retina (NR, *pink*) and ciliary epithelium (CE, pigmented—*grey*, non-pigmented—*green*). The inner layer of cells gives rise to the NR, while the outer layer gives rise to the RPE. The region in the middle, between the NR and RPE, gives rise to the CE, as seen in the right image. The highlighted square in the top right image shows what regions are found in the ‘ciliary margin,’ which includes both CE and the CMZ (CE = ciliary epithelial cells, CMZ = ciliary/circumferential marginal zone). This eye representation is typical of what a teleost fish/*Xenopus* eye looks like. The bottom right image shows the ciliary margin of species, such as mammals (e.g., mice), with more extensive CE development. Morphology and development can vary between species, but this is meant to highlight the variability that exists. (**B**) Summary of the characterization of CMZ cells across different organisms. *Green* checkmarks indicate that data supports the presence of these features, *magenta* ‘X’ indicates that data suggests these features are not present, and ‘?’ indicates that the data is insufficient to make a conclusion. Abbreviations: CMZ = ciliary marginal zone, RCJ = retinociliary junction, CM = ciliary margin, CB = ciliary body and CE = ciliary epithelium. A question mark is used when not enough evidence to date has been found to conclude the presence of a CMZ-like region.

The identity of cell types and regulatory mechanisms required to build retinal tissue in the embryo are well known, but are similar mechanisms capable of maintaining retinal tissue later in life? How does this knowledge of early retinal development inform our understanding of the potential for ongoing growth in the mature retina? Early in development, the retina is formed from specified tissue of the neuroectoderm that undergoes morphogenetic changes to form the optic vesicle. The optic vesicle invaginates to form a two-layered tissue of neuroepithelial precursor cells referred to as the optic cup, where cells found in the inner layer give rise to the neural retina (NR), and cells in the surrounding tissue form the retinal pigmented epithelium (RPE). At the peripheral edge of the optic cup where these two layers meet, the precursor cells adopt a ciliary epithelium (CE) identity and contribute to the formation of the ciliary body (CB) and other anterior structures of the eye (i.e., iris) [1] (Figure 1A). The neuroepithelial precursors specified to become the NR exhibit stem cell properties of self-renewal and multipotentiality, generating progeny that eventually differentiate to give rise to all seven retinal cell types organized into a tri-laminated structure. These NR-derived cells are often referred to as ‘retinal progenitor cells’ (RPC). In contrast, the neuroepithelial precursor cells specified to become RPE appear to have more limited proliferative potential and only generate a single distinct cell type—cuboidal pigmented epithelial cells [2]. Thus, despite a common origin of the NR, RPE and CE from the neuroepithelium of the optic cup, the precursor cells in these three main regions of the developing retina adopt distinct identities in vivo and generate unique lineages contributing to a diversity of neuronal and non-neuronal cell types. Whether similar neuroepithelial precursor cells persist in the mature retina as a dedicated population of stem-like cells has been debated, but as discussed in this review article, evidence from different vertebrate species suggests that this is the case. Therefore, neuroepithelial precursors are crucial for the initial development of the vertebrate retina but may also have a function in the maintenance and plasticity of mature retinal tissue.

Post-embryonic retinal growth varies widely between species. For example, in mammals, increases in ocular diameter from birth to adulthood can range from 25–250%, and this corresponds to an increase in the retinal surface area [3]. However, for most mammalian species that have been examined, neurogenesis in the NR ceases within the first 1–2 postnatal weeks, suggesting that any change in post-embryonic retinal growth that is associated with eye growth is mostly driven by non-neurogenic processes, such as stretching of peripheral non-sensory retinal tissue [3]. For example, human eyes are morphologically fully developed by 2–3 years of life, which is equivalent to postnatal day 21 in mice [4]. However, the overall shape of the eye normally continues to elongate or shorten into adolescence to properly focus the light on the retina due to the process of emmetropization, which has largely been attributed to biophysical changes to the overall globe of the eye rather than cell proliferation [5]. In contrast, other species, including teleost fish and amphibians, exhibit continuous growth in the retina over their entire lifetime [6,7]. Corresponding to this growth, these species have a clearly identifiable source of actively proliferating neuroepithelial cells at the periphery of the NR, in a region termed the ciliary or circumferential marginal zone (CMZ). Spatially the CMZ represents the very far tip of the NR, adjacent to the lens, sandwiched between the ends of where the RPE and NR converge, but are distinct from and do not include the peripheral RPE or CB (Figure 1A). Mammals have extensive development of the CB, and thus, regions describing the inclusion of cells from the peripheral RPE or CB, made of pigmented or non-pigmented epithelial cells, respectively, are instead often referred to broadly as the ciliary margin. 

Due to the proliferative nature of cells in the CMZ in some species beyond early post-embryonic stages and during adulthood, this region has long been speculated to represent a bona fide retinal stem cell niche that may be conserved across species. Other cell types of the eye, including the RPE, CE and Müller glia, have also been found to exhibit stem-cell-like characteristics in some organisms [6,8,9,10], but the peripheral NR shows the most consistent evidence of a stem cell niche, considering its spatial restriction and continuous growth activity in many vertebrates. Here, we consider the putative conservation of the CMZ across vertebrate species and the proliferative parameters, multipotency and growth mechanisms of these cells to understand how they behave in vivo under homeostatic or altered physiological conditions. We review our current understanding of the known signalling and molecular factors that regulate the activity and size of the CMZ and how disruption of this regulation could lead to dystrophic eye growth conditions, such as myopia. Lastly, we consider the capacity of CMZ cells for repair or prevention of retinal pathologies by comparing regenerative mechanisms and capabilities in species that show high CMZ activity. A better understanding of the properties of CMZ cells in the mature retina, including the identity of putative retinal stem cells (RSCs) and the mechanisms that regulate them, will enable important insight into how an endogenous generative tissue compartment can adapt to altered retinal physiology and potentially even restore vision loss caused by retinal degenerative conditions.

## 2. Multi-Species Comparison of an Identifiable CMZ or ‘CMZ-Like’ Region 

For decades, the retina of fish and amphibians has been known to continuously grow due to a maintained population of proliferating cells in the CMZ, which remains active through most of the organism’s life [6,7,11]. Thus, one of the hallmarks of a CMZ is the presence of a proliferative population of cells in vivo that can be easily identified using protein expressions, such as proliferating cell nuclear antigen (*PCNA*) or the ability of CMZ cells to incorporate a thymine analog, such as BrdU/EdU, into their DNA during the S phase of the cell cycle [6,12]. However, this metric has its limitations since quiescent cells, defined as being in extended periods of proliferative inactivity, may not be labelled and identified using these techniques. Therefore, in addition to proliferation markers, genes known to be expressed in embryonic neural stem and progenitor cells and associated with CMZ cells of the mature retina in fish and amphibians, including *Rx*, *Chx10/Vsx2*, *Sox2* and *Pax6*, help to further define the CMZ niche [6,7]. Different vertebrate species may exhibit non-proliferative cells in the CMZ or CMZ-like regions in vivo despite containing markers associated with proliferative CMZ cells (including the CE cells of the CB) and can instead be evaluated for proliferation potential by in vitro assays, such as the neurosphere assay, that evaluate the ability of isolated and cultured cells to clonally proliferate and give rise to multilineage fated cells [13]. 

Using these parameters, evidence of a CMZ-like region across different species has been mixed (summarized in Figure 1B). Other fish species, such as catsharks, show significant reductions in retinal cellular density in the mature retina, indicating reduced retinal cell production over time and no evidence of a maintained CMZ-like region that expresses proliferating markers [14]. This is in contrast to the margin of the juvenile catshark retina, which shows a clear expression of genes involved in proliferation (i.e., *Sox2*, *PCNA* and *pH3*) and neurogenesis [14]. This does not necessarily rule out the presence of proliferative CMZ-like cells in the mature retina, but it at least suggests that if present, these cells adopt quiescent features for significantly long periods of time. Interestingly, cave-dwelling cavefish, which initially form an eye primordium before suspending its development and initiating its degeneration, have an identifiable population of proliferating CMZ cells [15]. These CMZ cells remain actively proliferative even after eye growth is diminished, indicating that retinal degeneration in cavefish is not associated with the reduced proliferative capacity of the CMZ [15]. This is surprising since the presence of the CMZ has largely been correlated with species that exhibit extended periods of retinal growth into adulthood. For example, comparisons across squamates show a link between postnatal eye growth and differences in the presence and activity level of a proliferative peripheral region, referred to as the retinociliary junction (RCJ)—a region closely resembling the CMZ found between the NR and CB [16]. In fact, squamates show a visible depletion of the proliferating cell populations from juvenile to adult stages, as eye growth rates drastically decrease [16]. Another study in reptiles examining the adult retina of anoles, queen snakes, garter snakes, painted turtles and snapping turtles show that all have an expression of the multipotent progenitor markers, *Pax6* and *Sox9*, in cells at the margin, but only turtle retinal margins have *PCNA+* proliferating cells [17]. Comparatively, birds, including chicken and quail, both show an active proliferating pool of cells at the CMZ as they can be labelled with BrdU and also positively label with progenitor markers, such as *Chx10*, *Pax6* and *PCNA* [18,19,20,21]. These studies highlight that different species exhibit some shared CMZ characteristics, but proliferation varies widely, especially with the increasing age of the organism. 

In other vertebrate classes, such as mammals, the identification of a CMZ has been difficult due in part to their severely reduced/lack of proliferative capability in vivo in the mature retina. Moreover, the space between the NR and the CB contains so few cells (as mentioned above) that it appears a similar CMZ region has almost completely diminished in the mature retina. Instead, the CB tissue at the peripheral margin is much more elaborate and has been shown to house cells with proliferative potential [22] (Figure 1A). Comparative analysis in the mature retina of marsupials, opossums and mice showed that marsupials and opossums show sparse BrdU labelling in the CB but not in the CMZ, whereas no evidence of proliferating cells could be found in mice [18]. Instead, experiments that isolated cells from the CB from adult mice identified pigmented CE cells at the margin capable of proliferating in vitro [23,24,25]. This indicates there may be retinal cells with proliferative potential at the margin, but in vivo, proliferation is rare. These marginal cells are not technically part of what would normally be considered a CMZ, but they do represent a CE cell type that may be capable of producing multilineage NR cells, at least under experimental conditions. Recent transgenic mouse lines labelling *Msx1*-positive cells [26] and *Zic2*-positive cells [27] have been found to label a subset of CMZ cells in the embryonic retina. However, in the postnatal retina, these CMZ cell types are nearly absent, with *Msx1*-positive cells becoming limited to CE cells in the CB [27]. *Msx1* and *Zic2*-positive cells in the embryonic retina incorporate EdU and label with other proliferation and progenitor cell markers (i.e., *CyclinD2*, *Ki67*, *Sox2* and *Chx10*) in vivo [26], but their proliferation is limited postnatally, suggesting CMZ cells in mice lose their proliferative characteristics in vivo relatively soon after birth under normal conditions [26,27]. Interestingly, the proliferation of cells in the peripheral margin of the rat retina has been shown to increase in a degenerative model of retinitis pigmentosa [28], suggesting that these marginal cells may only proliferate under very specific conditions rather than being used as a source of continuous growth. Furthermore, the identification of molecules to enhance proliferation in mice showed that glucocorticoid agonists could stimulate CE proliferation in the adult retina in vivo [29]. These studies suggest proliferative cells likely exist at the margin in most vertebrates, but there are differences in their tissue localization and neurogenic activity in vivo.

Few studies have suggested that cells with proliferative capability are similarly found in the human adult peripheral retina. Assessing the expression of different proliferative markers indicates the marginal cells can be distinguished from neural retina due to the differential expression of *Nestin*, *CRALBP*, *Sox2*, *Chx10* and Shh and glial-like features [30]. In agreement, cells isolated and grown in culture from the peripheral human retina are largely *Nestin*+ with radial glia markers, glutamine synthetase and *GFAP*, capable of producing differentiated cells of different morphologies and correspondingly varied gene expression [31]. Intriguingly, in human eyes with proliferative vitreoretinopathy, a retinal detachment condition that induces RPE proliferation, epithelial cells in the CB and glial-like cells at the peripheral retina are also stimulated to proliferate [32]. To further study the development of a stem cell niche in the human retina, self-organizing human retinal tissue on 3D culture can re-create the stratified neural retina with a ciliary margin-like stem cell niche displaying proliferative cells with organized expression domains of factors typically seen in CMZ cells, such as *Rx*, *Chx10* and *Sox2* [33]. While this in vitro model does not fully recapitulate how the human eye develops and does not necessarily represent features present in the mature retina, it highlights an important experimental approach for gaining new insight into human retinal development, in general, and more specifically, the formation of putative neurogenic niches in mature retinal tissue.

The studies described above suggest that a CMZ or CM-like region is likely to be a conserved feature of the mature retina in most vertebrate species (Figure 1B). However, the source of the proliferative cells remains to be fully understood. Mammalian species appear to have a CE-like proliferative cell type found in the CB, whereas teleost and amphibian proliferative cells are part of the peripheral NR in what is defined as a CMZ. Based on a few markers and proliferative and multipotent potential, the different molecular properties underlying these distinct RSC-like cells are unclear. This research would benefit from a larger genome-wide analysis of chromatin, epigenome and transcriptome to characterize the comparative genetic landscape of these cells [34,35,36]. Regardless of the variation in cell type, the extent to which CE/CMZ cells are present and proliferate in vivo appears to be related to species adaptations and are dependent on age (i.e., juvenile vs. adult retina) and context (i.e., growth, injury or molecule stimulation). Moreover, the fact that the presence of a CMZ-like region does not necessarily correlate with continuous retinal growth suggests that tissue maintenance and/or plasticity in response to changes in physiology (uncoupled from ‘growth’ per se) may be an integral function of this specialized region of the vertebrate retina. 

## 3. Distinct Cell Types within the CMZ Niche and Their Proliferative and Multilineage Potential 

Proliferating cells are found in the CMZ, but do these represent true stem cells? To be considered a true tissue-specific stem cell, most researchers agree that the stem cell must meet these three criteria: (1) reside in the niche over long periods of time, (2) self-renew and (3) give rise to multiple cell types in the tissue [37]. Neural stem cell niches that are found in other regions in the central nervous system, including the mammalian forebrain subependymal zone (also known as the subventricular zone (SVZ)) and the hippocampal subgranular zone (SGZ) [38], are typically composed of different subclasses of proliferative cell types. In the SVZ and SGZ niches of the brain, the neural stem cells are typically defined as radial-glial-like or neuroepithelial-like cells that give rise to a highly proliferative intermediate progenitor cell type referred to as a transit-amplifying cell [39]. The retinal stem niche model can be described in a similar hierarchical order. For example, RSCs are described as long-term resident cells with proliferative potential and self-renewal capability, whereas RPCs refer to highly proliferative intermediate cell types that amplify the progenitor pool before ultimately differentiating and leaving the niche. To describe a proliferating CMZ cell as an RSC or RPC, it is necessary to track the proliferative and cell lineage potential over extended periods of time. Most of these studies come from fish and amphibian laboratory models and have been instrumental in defining the characteristics of CMZ-mediated retinal growth. 

**Figure 2:**
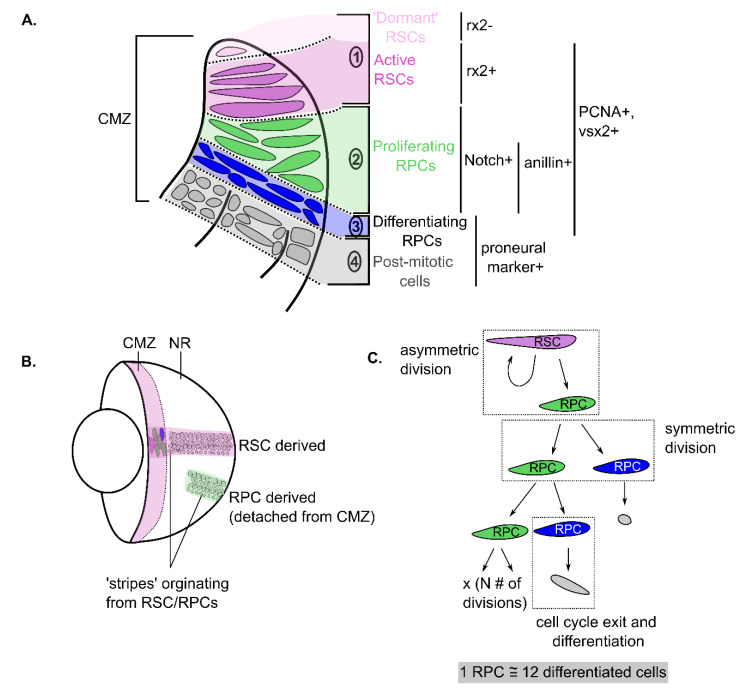
Retinal stem and progenitor cells’ characteristics in the CMZ of teleost fish and *Xenopus*. (**A**) Schematic of the four main compartments of the CMZ niche. The first region at the tip (1) contains dormant and active retinal stem cells (RSCs) (*pink*) that are either *rx2-* or *rx2+*. In the second region are highly proliferative retinal progenitor cells (RPCs) that are distinguished by expression of a different subset of markers (i.e., Notch and *Anillin*). The third region is composed of RPCs poised to differentiate since they co-express proliferating and pro-neural genes. All actively proliferating RSC/RPCs express markers for proliferation, including *PCNA* and *vsx2*. In the fourth region are post-mitotic cells that have differentiated. This schematic is adapted from those seen in [6,7]. (**B**) Schematic of the whole eye showing the CMZ region labelled in *pink* in an annulus adjacent to the lens. Labelled populations of cells derived from RSCs produce continuous stripes from the CMZ (named ArCoSs), whereas stripes produced from RPCs produce disconnected patches called terminated patches. This schematic is adapted from data visualized in [41,46,48]. (**C**) Example of the different events occurring in the proliferation and differentiation of RSCs/RPCs in the CMZ. An asymmetric division gives rise to two different daughter cells. An RSC that asymmetrically divides gives rise to one RPC and one RSC to maintain self-renewal. Conversely, a symmetric division results in the progeny of the same identity (i.e., two RPCs). RPCs will eventually stop proliferating and differentiate after a certain number of divisions, on average producing 12 differentiated cells. *Pink* cells = RSCs (zone 1 cells), *green* cells = proliferating RPCs (zone 2 cells), *blue* cells = RPCs committed to differentiation (zone 3 cells) and *grey* cells = differentiated cells. Adapted from data presented in [42].

The CMZ of teleost fish and amphibians can be broken down into subsections based on the expression of multiple factors [6,11,40] (Figure 2A). At the very tip, known as the ‘peripheral CMZ,’ is the subregion thought to be the specific location of resident RSCs that proliferate slowly and divide asymmetrically to ensure continuous self-renewal [41,42]. The RSCs have been associated with the expression of markers, including *rx*, *six3*, *pax6* and *vsx2* in zebrafish [6] and *Xenopus* [7]. These RSCs are typically found within a small cell cluster at the CMZ peripheral tip and stay attached to the same spatial location where they originated [40,42]. These RSCs give rise to RPCs that reside in the next adjacent subsection, also known as the ‘central CMZ’ [6,41]. These RPCs, although sharing expression of factors with RSCs, also express signalling molecules associated with Notch signalling [6,7]. Despite some differences in expression, distinct identification of RSCs versus RPCs has been challenging [43]. New markers or transgenic-labelling of CMZ populations have emerged, and recent molecular labelling has shown that RSCs are specifically labelled by *rax/rx2* [44], whereas the F-actin binding protein, *anillin*, labels late RPC populations in fish species [45]. A third CMZ subregion contains committed RPCs that express multiple different proneural genes, and these cells will ultimately produce the post-mitotic cells present in the fourth and final zone found closest to the border with the central retina [6,11]. These multilineage differentiated cells eventually move centrally into the retina as the retina continues to grow, resulting in concentric additions of new cells.

Although the different types of CMZ cells can be generally distinguished by different sets of molecular markers, lineage tracing of individual CMZ cells over time has confirmed the lineage relationship between them in vivo. Early transplantation studies [46,47] and, more recently, genetic-based lineage mapping [26,41,42,48] have shown that according to its spatial organization, the CMZ is indeed composed of both RSCs and RPCs. Continuously labelled clones tracked over time in the growing eye of teleost fish form characteristic labelled ‘stripes’ of displaced cells demarcating their origin and destination along the clone [41,46,48]. Two different types of stripes/patches are commonly observed, including those that continuously label from the CMZ all the way into the central retina, termed arched continuous stripes (ArCoSs) [41,46,48], and those that label smaller stripe in the central retina that have become disconnected from the CMZ, termed ‘terminated patches’ [48] (Figure 2B). Stripes connected to the CMZ, such as ArCoSs, are believed to originate from dedicated RSCs that never leave the margin, whereas disconnected patches likely originate from RPCs that proliferate a limited number of times before leaving the niche and differentiating. In agreement with the localization of these stem cell populations, ArCoSs are more likely to originate from the peripheral CMZ, whereas terminated patches commonly originate from the central CMZ [6,48]. At the very tip of the zebrafish CMZ, however, cells that lack *rx2* but positively label with retinal stem cell marker, *mz98* [43], have also been identified [40] (Figure 2A). These cells remain at the CMZ tip over long periods of time without proliferative activity seen from 3–14 day old zebrafish, suggesting they behave differently than the previously studied RSCs [40]. Unfortunately, the fate of these cells and whether they represent a ‘quiescent’ stem cell population perhaps similar to those found in mammals is unknown. Future studies should be focused on investigating the properties of these cells in more detail. Due to their quiescent nature, long-term lineage studies may be required to probe their potential. Nonetheless, CMZ generated stripes commonly contain all retinal cell types, distributed in a compact column among the tri-laminated retinal layers indicating both RSC and RPC CMZ cells are likely to be multipotent, but RPCs have a more limited proliferative capacity [41,46,48]. 

It is important to consider how the proliferation properties have adapted to maintain these cells in the CMZ niche without depletion over time. As new differentiated retinal cells are produced into the mature retina, proliferating CMZ cells can either be continuously displaced and replaced by chance, which is a form of neutral competition and drift, until one clone dominates the CMZ niche, or all clones can be maintained, if RSCs invariantly divide asymmetrically to maintain a clone in the niche [41,48]. This behaviour depends on the choice to divide symmetrically to produce two daughter cells of the same identity or asymmetrically to produce two daughter cells with differing identities [49] (Figure 2C). In a deterministic model, stem cells invariantly divide asymmetrically to produce a stem cell and progenitor daughter cell, which helps maintain individual clones in the stem cell niche. RSCs in the peripheral CMZ seem to follow this pattern, as they slowly preferentially divide asymmetrically, even under retinal injury, to maintain a constant number of RSCs over time [40,41,42] (Figure 2C). RSCs, however, can increase in number as the retina proportionally grows, suggesting less frequent symmetric divisions can also occur to increase the pool of active RSCs [41]. Conversely, RPCs appear to divide variably between symmetric and asymmetric divisions a limited number of times before ultimately differentiating (Figure 2C). As they differentiate, they leave the CMZ niche, allowing other newly generated RPCs to take their place [42,48]. RPCs within the CMZ appear to divide to produce, on average, 12 differentiated cells per RPC [42]. Interestingly, this is quantitatively similar to embryonic RPCs [50], suggesting shared RPC behaviour between early and mature RPC populations. This contrasts with RSCs, which appear to have adopted a different pattern of proliferation congruent to the ability to maintain a stable clonal population in the far peripheral CMZ. 

Comparatively, the characterization of the stem cell properties, lineages and multipotency of identified ‘RSCs’ in other species, particularly mammals, is less consistent [51,52]. Initial reports of isolated, cultured CE cells from the adult mouse CB suggested that they demonstrate properties of RSCs in vitro: proliferation, self-renewal, gene expression and retinal multilineage potential [23,25]. Subsequent studies have reported differences in the ability (or inability) of these isolated adult mouse CE cells in vitro to produce photoreceptors [24,53,54]. However, in vitro culture conditions can have a significant impact on the differentiation of clonal CE-derived cultures, suggesting that when conditions are relatively optimal, multilineage potential in vitro can be observed [23,28]. In vivo, *Msx1+* and *Zic2+* CMZ-like cells in the mouse retina have been shown to produce multiple neural retinal lineages in the embryonic retina [26,27], but these same lineages fail to contribute to neurogenesis in the postnatal eye [26]. Interestingly, lineage tracing in vivo and using in vitro culture studies have recently identified *c-Kit*+ RPC-like cells distributed instead throughout the neural retina, which do not co-label with other differentiated retinal cells, [55,56], and can give rise to retinal cells in the inner nuclear layer (INL) and outer nuclear layer (ONL), including both neural and glial populations, postnatally [56]. These *c-Kit*+ cells, although significantly reduced in the older retina, are still present in up to 57-week-old mouse retinas [56]. The presence of these apparently undifferentiated cells in the mature NR contradicts reports that failed to identify stem cells from older NR tissue [23,25,57]. This highlights the enduring challenges of identifying RSC-specific properties in vivo in adult mammals. Overall, these and other published studies suggest that species with a morphologically defined CMZ niche contain RSCs that have maintained their proliferative and multipotent characteristics to give rise to RPCs that eventually differentiate and emigrate from the niche to contribute new cells to the mature retina. In contrast, species without a defined CMZ niche harbour CE cells with the potential to demonstrate RSC properties in vitro but with significantly limited in vivo neurogenic activity.

## 4. CMZ RSCs as a Source of Neural Retina and Retinal Pigmented Epithelial Cells?

**Figure 3:**
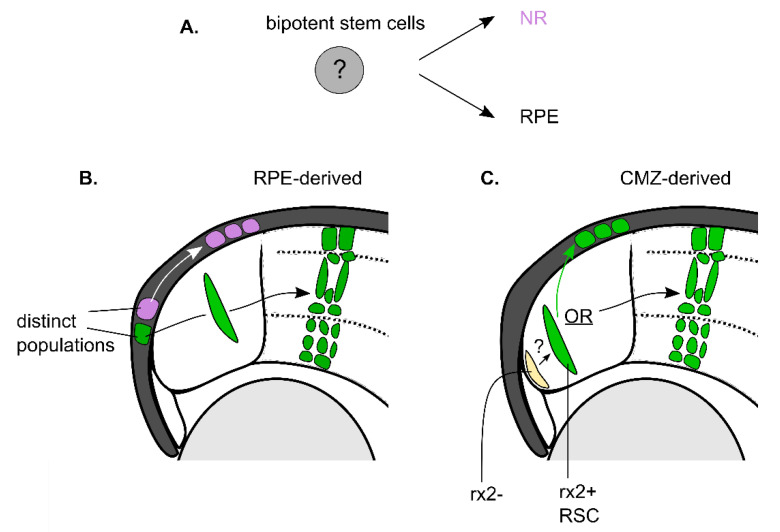
RPE and NR cells are generated from distinct stem cell populations at the margin. (**A**) Schematic of what a ‘bipotent’ stem cell represents—a cell capable of producing both neural retinal cells (NR) and retinal pigmented cell (RPE) lineages. (**B**) Cells in the RPE of the teleost show are shown to contain cell populations that give rise to the RPE or the NR [46]. However, the RPE and NR cells have distinct cell origins, suggesting a bipotent stem cell does not exist in the RPE. *Pink* cells (that produce RPE) and g*reen* cells (that produce NR) represent distinct stem cell populations. (**C**) CMZ *rx2+* cells (*green* cell in the CMZ) can give rise to RPE or NR but not to both simultaneously, suggesting these cells are also not bipotent but become committed to one of the lineages [44]. The *rx2+* cells appear to choose between the two fates. Therefore, all cells are represented as *green* to display the different paths an *rx2+* cell can choose. At the far periphery of the CMZ are *rx2-* cells (*light yellow*), and it currently remains unknown whether they can contribute to the production of the *rx2+* RSC population (this uncertainty in represented by a ‘?’).

Lineage tracing experiments demonstrate that proliferating CMZ cells predominantly generate populations of NR cells. However, do these cells also have the potential to produce RPE or CE (Figure 3A)? Due to several interesting lineage relationships known between the RPE and NR, it is of interest to know whether the stem cells of these two lineages are common or independent. As described above, during eye development, an initially common pool of neuroepithelial precursor cells in the optic vesicle becomes specified and committed to either the NR or RPE lineages [1,46,58]. Early lineage tracing of zebrafish embryonic retinal cells shows that RSCs of the CMZ and pigmented cell progenitors arise from the division of a subclass of migrating “bipotent” precursor cells present in the developing optic vesicle [40]. This lineage migrates to the peripheral edges of the optic cup and contributes to the formation of both populations at the margin. Moreover, lineage tracing of RPE progenitor populations in the developing zebrafish eye shows that these progenitors can contribute to the *rx*-negative, *mz98*-positive population of inactive CMZ cells found at the peripheral tip [40]. However, this analysis was limited to the developing eye and therefore is limited in its interpretation for the stem cell populations in the adult retina. Analysis of genetic mutants that disrupt either the RPE or CMZ agrees with the notion that RPE and CMZ fates are intertwined. Mutants that show compromised structure of the CMZ are often accompanied by overgrowth of the peripheral RPE [59,60], and conversely, loss of RPE can be accompanied by ectopic production of NR [8,61]. These studies suggest the two tissues are intimately tied in their development and rely on a common lineage relationship to produce the two dedicated stem cell populations at the retinal margin.

The link between the RPE and NR is clear in the developing retina, but does the mature retina show a similar relationship? In the mature retina, the RPE and NR are seen to grow at similar rates in a continuous growing eye. Studies in teleost fish show that, similar to the NR, RPE generates continuous stripes or ArCoSs [46], indicating similar growth dynamics from a pool of putative RPE progenitors. Interestingly though, the NR displays more consistent steady growth than the variable, intermittent growth of the RPE, suggesting a model where the RPE integrates inducive signals from the NR to match and coordinate growth [48]. Rather than relying on a common progenitor for this matched growth, these populations appear to arise from distinct, individually regulated clones of marginal cells that are committed to either produce the NR or RPE lineage [41]. This is because transplanted pigmented GFP+ marginal cells are seen to give rise to NR or RPE but rarely display overlapping RPE and NR ArCoS stripes, as would be expected if a common stem cell was producing both lineages [46] (Figure 3B). This is in contrast to earlier lineage tracing in *Xenopus* that suggested single clones of CMZ cells could contribute to both NR and RPE lineages [47]. The presence of pigmented cells at the retinal margin with proliferative potential in zebrafish is reminiscent of the fact that pigmented CE cells in the mouse and human retina have RSC properties [23,25,57]. Moreover, studies in amphibians (i.e., *Xenopus laevis* and newts) have additionally shown that RPE can transdifferentiate or reprogram to give rise to NR after complete retinoectomy [8,9,62], showing extreme cases where NR are derived from RPE cells. The common feature in these studies is that the source of new cells is a pigmented cell type (RPE or CE), but whether these pigmented cells represent a resident, uncommitted stem-cell-like subpopulation capable of producing NR and RPE, similar to the common neuroepithelial cells in the developing optic vesicle, is unknown. Conversely, is it also possible that the RSCs in the CMZ give rise to NR and RPE? Recently, a population of *rx2*-positive RSCs in the CMZ has been shown to generate clones entirely of either RPE or RPC progeny but apparently never contribute to both lineages [44] (Figure 3C). Whether subpopulations of *rx2*-positive cells exist or whether there are instructional signals that facilitate the production of distinct lineages (i.e., RPE vs. RPCs) is unknown. Overall, these fascinating observations provide some of the most compelling evidence to date for the presence of possibly heterogeneous populations of stem cells at the retinal margin that maintains tissue growth. However, long-term genetic lineage tracing is still required to determine the lineage potential of these stem cells, particularly in the adult retina. Future experiments that focus on clarifying the identity of these cells (e.g., distinct gene expression profiles) and utilizing these markers for lineage tracing experiments to help determine their lineage relationships and in vivo potential will be critical for understanding the role of stem cells in the maintenance of retinal tissue. 

## 5. Maintaining the CMZ Cells through Transcriptional Regulation

**Figure 4:**
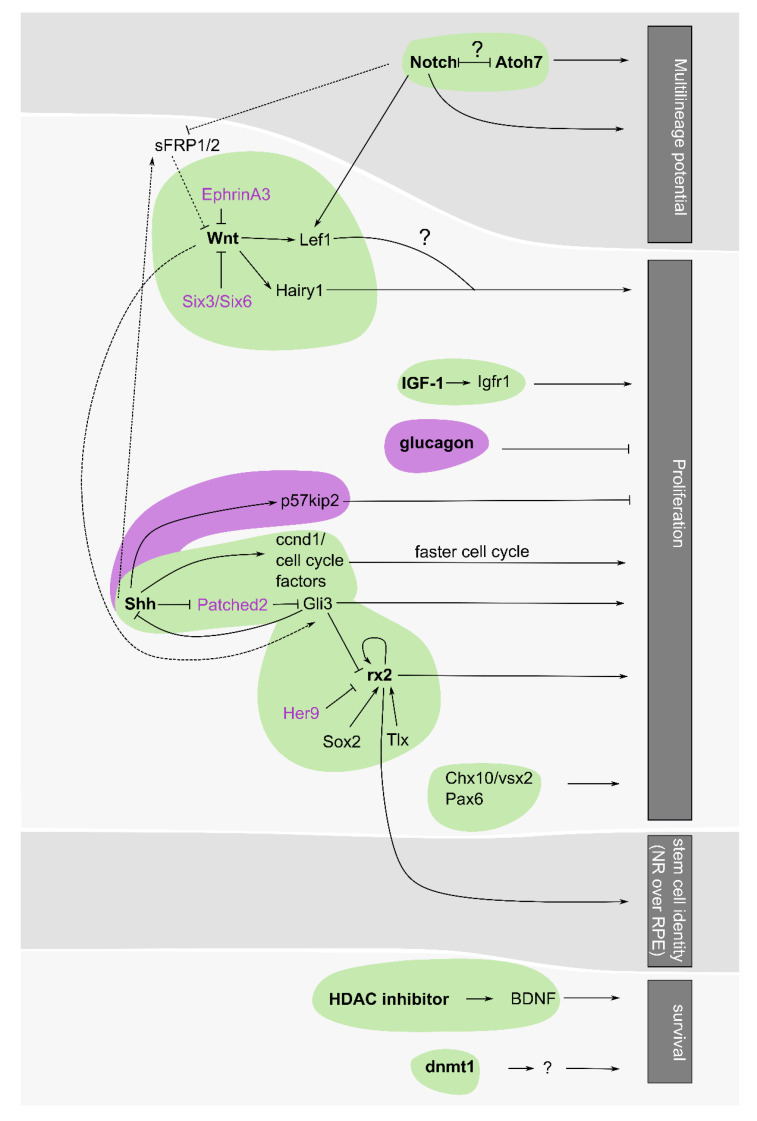
Transcription and signalling pathways regulate and limit proliferating RSC/RPCs to the CMZ. A summary of the complex regulation and inter-connected regulation of the CMZ niche—a region where multiple pathways collide. Shown is the known effect of transcriptional regulation (HDAC inhibitors, *dnmt1*, *Chx10/vsx2*, *Pax6*, *rx2*, *Six3/Six6*, *Atoh7*) and signalling pathways (Shh, glucagon, IGF-1, Wnt and Notch) presented in this review and how they converge onto the different aspect of RPC regulation- survival, stemness, proliferation and multilineage potential (*grey* categories presented on the right). The *green* circles highlight different hubs of signalling/transcriptional networks that positively regulate RSC/RPCs. *Pink* circles, however, negatively regulate RSCs/RPCs. Similarly, factors with *pink* lettering are used to represent factors that negatively regulate their downstream targets. Arrowheads at the ends of the line indicate a positive regulation, whereas blunt line ends represent negative regulation. Solid lines indicate linear relationships, whereas dashed lines indicate relationships between the hubs (i.e., between Shh and Wnt signalling or Notch and Wnt signalling). If any relationship is unknown or questioned due to inadequate evidence, a question mark is used to represent this uncertainty.

There is considerable interest in understanding the molecular underpinnings that regulate stem and progenitor cell behaviour at the retinal margin, including the transcriptional networks that control the maintenance and proliferation properties of CMZ cells (summarized in Figure 4). The expression of genes encoding transcription factors in the CMZ of *Xenopus* and zebrafish [6,7], for example, has been instrumental in laying the foundation for understanding the molecular regulation of CMZ neurogenesis. However, it has been challenging to disentangle the key factors involved in regulating RSCs versus RPCs in species with a well-defined CMZ or to gain insight into the similarities and differences in the cell types present at different ages (i.e., embryonic vs. post-embryonic). Comparison of single-cell RNA-sequencing data from embryonic and post-embryonic precursors in zebrafish suggest that the expression patterns and progressive states from RSCs to RPCs to differentiated cells from the CMZ are largely recapitulated in their embryonic RPC counterparts [63]. Whether this is true for other species is unknown, but it suggests that CMZ RSCs utilize similar genetic programs as their younger equivalent. However, functional experiments to test this idea can be challenging. For example, zebrafish germline loss-of-function gene mutations affecting the retina usually results in defects in early RPCs during retinal development [2,64]. We have shown using early transient loss-of-function experiments that the transcription factor, *dmbx1a*, is involved in regulating the cell cycle exit of embryonic RPCs [65,66]. Recently, we generated *dmbx1a* germline mutants, which show phenotypes mainly in post-embryonic retinal growth, in part due to impaired maintenance of cells produced from the larval CMZ (Miles and Tropepe, unpublished data). Therefore, our study, as well as others that describe mutations that predominately affect the post-embryonic CMZ, suggest that to some extent, different regulatory networks may be required even though there are similar genes expressed in the cells across the lifespan [58,60]. 

In recent years epigenetic factors have emerged as key regulators of adult neural stem cells [67,68], although only a few factors have been implicated in CMZ cell maintenance so far. Histone deacetylase inhibitors (HDACi), for example, have been seen to rescue significant amounts of cell death observed in the CMZ of *dying on edge (dye)* zebrafish mutants, which have a mutation in the vacuolar ATPase subunit gene, *atp6v0e1* [69,70]. Mechanistically, it was shown that HDACi rescues CMZ survival via upregulation of brain-derived neurotrophic factor (BDNF) and that treatment with a BDNF mimetic is sufficient to similarly rescue these defects in the mutant [69]. This study suggests that histone acetylation may have an important role in CMZ cell neuroprotection. Other studies have similarly shown that HDAC1 regulates genes important for the dedifferentiation of Müller glia during retinal regeneration in zebrafish [71]. DNA methyltransferase 1 (*dnmt1*), expressed in the CMZ of zebrafish, has also been shown to be important for CMZ cell survival. *dnmt1* knockout in zebrafish results in a significant elevation in cell death in the CMZ, aberrations in CMZ cell cycle gene expression and decreased cell proliferation and differentiation from RSCs [72]. Interestingly, in mice, *Dnmt1* is expressed in CMZ cells, suggesting a possible conserved role in the mammalian retina [73]. Other epigenetic factors have only be described in embryonic RPC proliferation and survival, including *G9a* [74,75], *Ezh2* [76] and *PRC2* [77], but have not been studied in detail at older stages. 

Multiple different families of transcription factors have been seen to be crucial for the proliferation, identity, multipotency and differentiation of RPC in the developing retina [2,78,79], but the factors specifically involved in CMZ regulation are less well described. The CMZ expresses known RPC regulating transcription factors, including *Rax* genes (*Rx1*, *Rx2*), *Chx10/Vsx2* and *Pax6* [6], which are known to play crucial roles in RPC proliferation in the developing retina [79,80,81]. As mentioned above, *Rx2* labels the most peripheral CMZ cells in the medaka retina, corresponding to RSCs capable of producing either complete lineages of RPE or NR [44], and its paralogs have also been found in the CMZ of other fish species and frogs [6,15,82]. When *rx2* is knocked out, RSCs favour the formation of RPE instead of NR, suggesting that *Rx2* is crucial for lineage determination [44]. In *Xenopus*, expression of the *rax* homologue in the dorsal CMZ is found to be autoregulated by *rax* binding to a regulatory element, UCE1, which is found in the *rax* coding region [82]. In medaka, a proximal regulatory element of *rx2*, pCRE, regulates *rx2* expression at the CMZ and is shown to be bound by four transcription factors, including *Sox2*, *Tlx*, *Gli3* and *Her9* [44]. *Sox2*, *Tlx* and *Her9* are known to be important regulators of neural stem cell maintenance and/or Müller glia activation in the regenerative response [71,83,84,85], suggesting shared networks exist between different kinds of stem cell populations. *Sox2* and *Tlx* promote *Rx2* expression, but overexpression of these two factors can promote re-initiation of proliferation in post-mitotic retinal cells [44]. *Gli3* and *Her9* instead inhibit *Rx2* expression in regions outside the CMZ to limit RSC proliferation in the CMZ [44]. Other *Hes/Her* transcription factors have similarly been found to be expressed in the CMZ and to be necessary to maintain the proliferative state and cell cycle kinetics of RSCs [86,87,88]. This network helps define how balanced expression of transcription factors is necessary to regulate core RSC genes (i.e., *Rx2*) to confine the proliferating region strictly to the CMZ. Given the different types of cells that have been identified in subregions of the CMZ, one major gap in our knowledge with respect to transcriptional regulation is the extent to which these transcription factors and epigenetic regulators affect distinct subpopulations of CMZ cells. In the future, conditional gene gain/loss-of-function approaches [89,90] in specific populations of RSC/RPCs would be highly desirable for investigating the in vivo regulation of specific CMZ cells in either affecting their proliferative potential, lineage potential/multipotency or survival. 

## 6. Microenvironment of the Niche Confines Proliferation to the CMZ

In addition to transcriptional regulators, the CMZ niche is regulated by different extracellular signalling molecules. These signalling molecules, including IGF, Shh, Wnt, glucagon and Notch and their effector genes, are expressed in CMZ cells and have been associated with the regulation of different aspects of cell behaviour (summarized in Figure 4). However, most of these factors have been implicated in controlling proliferation and appear to define and limit this proliferation to the CMZ. 

First, growth factors, such as insulin-like growth factor-1 (IGF-1), are shown to stimulate RSC proliferation in vitro or in vivo [52,62,91]. The IGF-1 receptor is expressed in the CMZ region of goldfish [92], and injection of IGF-1 has been shown to increase the proliferation of RPCs at the CMZ [93]. Recently, the IGF1 receptor (*Igf1r*) in zebrafish is also shown to regulate CMZ proliferation [94]. *Igf1r* activation decreases the cell cycle length in the CMZ, expands the progenitor population and results in the increased production of differentiated neurons to cause an overall increase in the size of the eye. However, the source of endogenous IGF in the retina is unclear.

The mature retina also produces sonic hedgehog (Shh) from retinal ganglion cells [95,96], where it signals to adjacent CMZ cell populations and regulates its proliferation. The exact effect of Shh depends on the species and context since different species have shown differing results. In *Xenopus*, Shh has been shown to promote cell cycle exit of RPCs and thus has been associated with overall inhibition of CMZ proliferation [95]. In this case, Shh promotes cell cycle exit of CMZ cells through activation of cell cycle genes, such as cyclins, resulting in reduced G1 and G2 phases that overall decrease cell cycle length and result in precocious differentiation [97]. Given that upregulation of cell cycle genes has conversely been seen to promote continuous proliferation [66,74], it is unclear how elevated cyclins alone explain this phenotype. Embryonic studies of RPC cell proliferation in zebrafish similarly suggest Shh can promote cell cycle exit through activation of cell cycle inhibitor *p57^kip2^* [98]. However, in the mature CMZ of zebrafish, Shh has instead been found to promote proliferation. Shh normally acts to inhibit its effector protein, *patched2 (Ptch2*), allowing transcription of *Gli* target genes [99]. Both *Ptch2* and *Gli1/3* are expressed in the CMZ of *Xenopus* and medaka [44,95]. In zebrafish, *ptch2* mutants exhibit ectopic and increased proliferation resulting in retinal dysplasia, suggesting Shh signalling promotes proliferation [100]. This is similar to the effect in the chicken retina, where Shh overexpression in *Pax6*+ CMZ cells induces *Gli3/Gli1* expression to promote CMZ proliferation [96]. Interestingly, expression of downstream target gene *Gli3* in zebrafish RPE cells prevents proliferation, but in the CMZ, it promotes proliferation, suggesting Shh signalling has opposing functions to restrict RSC/RPC proliferation to the CMZ [44]. 

Wnt ligand or effector gene (i.e., *Lef1*) expression is also observed in the mature CMZ in mice [101], *Xenopus* [95,102], zebrafish [103] and chicken [88], where it has been shown to promote CMZ proliferation. Interestingly, *Hairy1*, normally associated as a positive notch signalling effector, acts downstream of Wnt signalling in the chicken CMZ [88]. In fact, loss of Wnt signalling in the CMZ—resulting in decreased proliferation—can be rescued by the expression of *Hairy1* [88]. Upstream, *EphrinA3* has been shown to inhibit Wnt signalling, suggesting a possible mechanism for how CMZ proliferation is restricted in mammals [104]. *EphrinA2/A3* knockout mice show enhanced proliferation and regenerative capability of stem-cell-like cells, Müller glia [105]. Additionally, *Six3/Six6* transcription factors have also been seen to jointly inhibit Wnt signalling in the mouse retina [106]. In the far periphery where *Six3/Six6* expression is low, Wnt signalling is upregulated to promote a ciliary margin identity. However, in the more central CMZ, *Six3/Six6* is upregulated to inhibit Wnt-induced ciliary margin specification [106]. Therefore, the precise control of Wnt signalling at the margin helps to restrict proliferation to this domain. 

Glucagon has also been found to regulate CMZ proliferation. Amacrine cells in INL expressing glucagon and substance P, known as bullwhip or mini-bullwhip cells, project dense neuronal processes into the CMZ in the chicken retina and release peptides, GLP1 and glucagon [107,108,109]. Here, glucagon has been shown to suppress the proliferation of cells in the CMZ, whereas glucagon antagonist application promotes it, suggesting that glucagon-producing bullwhip cells negatively regulate CMZ proliferation in vivo under physiological conditions [109]. Although bullwhip cells have not been extensively studied in other organisms, the retinas of anoles, queen snakes, garter snakes, and painted and snapping turtles all contain bullwhip cells. Interestingly, however, dense accumulation of glucagon neurites at the CMZ was only seen in painted and snapping turtles, directly correlating with the reptilian species that have proliferating cells at the margin [17]. These observations suggest the intriguing possibility that species with an active CMZ utilize neuronal-dependent glucagon as an inhibitory mechanism to limit the extent of endogenous proliferation.

Notch and its downstream targets are also highly expressed in the CMZ in *Xenopus* [11], zebrafish [87], medaka [110] and mice [106], but instead of affecting proliferation, has an important role in the regulation of cell fate. Until recently, however, its role in the CMZ was largely uncharacterized. Interestingly, Notch, together with *Atoh7*, maintains the potency of stem cells by ensuring the full complement of retinal cells produced from the CMZ [110]. Notch and *Atoh7* expression in RPC populations of the CMZ are mutually exclusive, labelling distinct populations. This is consistent with studies in the mouse retina, which show that Notch signalling through *Hes1* represses *Atoh7* expression [111]. CMZ cells with active Notch signalling predominately give rise to INL cells, including Müller glia, amacrine cells and bipolar cells [110]. Accordingly, activation of Notch signalling in *Atoh7*-positive cells shifts the production of retinal neurons to an INL identity at the expense of ganglion cells. Conversely, Notch inhibition produces more *Atoh7* progenitors, significantly reducing the production of photoreceptors [110]. Therefore, the balance between Notch and *Atoh7* appears to ensure the full complement of cell types is generated from the CMZ. 

These various overlapping pathways suggest that the CMZ niche is a unique location in the retina where complex integration of signals can maintain the identity and function of the CMZ lineages. For example, in the *Xenopus* retina, it has been shown that Shh and Wnt signalling pathways can function antagonistically: Wnt activation affects Shh-dependent *Gli3*, and Shh signalling affects the Wnt factor *Sfrp-1* [95]. Moreover, Wnt and Notch signalling might work co-operatively to control proliferation and specification of RSCs since Notch signalling has been shown to regulate Wnt-dependent factors *LEF1* and *sFRP2* [112]. For some of these factors, the endogenous source of the signalling ligand is known (e.g., Shh from retinal ganglion cells and glucagon from bullwhip cells), but the upstream factors that control their expression and downstream effector mechanisms are not well understood. Insight into these pathways and a more direct comparison of the activity of these signalling pathways in the CMZ across a larger breadth of different species may help determine adaptations that mediate the physiological control of the CMZ (i.e., inhibition or activation) and why in certain species, RSC niches at the retinal margin remain largely quiescent. 

## 7. Dystrophic Eye Growth in the Post-embryonic Retina and its Relationship to the CMZ

In the early development of the retina, genetic perturbations affecting CMZ proliferation can influence the size and shape of the eye, resulting in conditions such as microphthalmia [2,74,78,79]. However, the effect that post-embryonic CMZ proliferation can have on the size and shape of the mature eye is significant. In well-known visual disorders, myopia and hyperopia, the axial length of the eye is lengthened or shortened, respectively, resulting in the defocus of light images on the back of the retina from close or distant objects [5]. These ocular growth conditions have traditionally been associated with the restructuring and stretching of the scleral tissue surrounding the retina [113,114], but defects in proliferation and growth from the CMZ could also potentially contribute to these disorders. In fact, changes due to pathological (high-grade) myopia can result in significant thinning of the retinal layers as the eye is stretched [113], and retinal restructuring and growth would be required to compensate for these changes. Form-deprivation [114,115,116,117] is an experimental treatment where diffusers are used to obscure and defocus vision is often used to mimic the changes occurring in myopia. Form-deprivation in primates has been shown to result in increased proliferation in the CMZ that is correlated with the extent of post-equatorial axial elongation [118]. Furthermore, mutations in the transcription factor *ZNF644* are associated with high-grade myopia [119,120], and in zebrafish, *znf644a/b* in complex with histone methyltransferase *G9a*, have been shown to regulate the proliferation of RPCs in the developing retina by repressing cell cycle and RPC genes [74,121]. In another zebrafish mutant example of myopia caused by a nonsense mutation in *Lrp2*, affectionately named bugeye, the CMZ RSCs deplete over time, suggesting disrupted maintenance of RSCs [122]. Conversely, *MFRP*, a gene associated with hyperopia [123], has interestingly been found to be expressed in the CE cells in zebrafish [124] and in mice, and mutations in *MFRP* are associated with retinal thinning of all retinal cell layers [125]. Changes in proliferation in this mutant have not been assessed, but this could be an interesting area for future study. *MRFP* is a frizzled-related protein implicated in Wnt signalling, which, as mentioned above, is a key regulator of CMZ proliferation [126]. It is also curious that another gene associated with hyperopia, called *Prss56* [123,127], is expressed in late RPCs in the developing retina but is also expressed in Müller glia cells and results in increased retinal thickness when mutated in mice [128]. These experiments suggest that changes in CMZ cell proliferation, and perhaps RSCs specifically, could contribute to myopia and hyperopia, resulting in altered retinal thickness and growth post-embryonically (Table 1). 

Considering the mechanisms that may lead to these changes, a genome-wide DNA methylation analysis examining the epigenetic mechanisms underlying the pathology of myopia found significant changes in key signalling pathways, including Wnt and IGF-1, two pathways we have already discussed that can influence CMZ proliferation [129]. In fact, form-deprivation myopia in mice increases the expression of Wnt signalling pathway proteins, including Wnt, *Frizzled5* receptor and *β-catenin* [130]. Given that Wnt signalling promotes RSC proliferation, these data suggest that the myopic phenotype could be mediated through increased CMZ activity, although this warrants further investigation. Additionally, treatment of post-embryonic chicken eyes with IGF-1 (along with FGF2) is sufficient to induce excessive ocular growth, in part due to stimulated proliferation of CMZ cells, ultimately causing extreme myopia [91]. This is in agreement with the evidence for IGF-1 promoting CMZ proliferation [94]. Shh signalling is also associated with myopia development, as form-deprivation causes increased expression of Shh and *Gli3* mRNA, and Shh treatment can increase axial length, whereas inhibition of Shh can reduce it [131]. Lastly, glucagon antagonist treatment or drug-induced death of ‘bullwhip’ amacrine neurons and their projections to the CMZ can also result in excessive ocular growth, similar to myopia [107]. Given the association of these signalling molecules with CMZ regulation, these studies all suggest that CMZ cell proliferation may underlie, at least in part, the plasticity of post-embryonic ocular growth and that dysregulated control of this proliferation may lead to hyperopia or myopia (Table 1). Further experiments that assess the extent to which the disruption of these signalling molecules leads to ocular growth abnormalities or rescue/exacerbate growth defects in reliable animal models of myopia/hyperopia through the regulation of CMZ activity will help inform the link between CMZ regulation and dystrophic ocular growth. 

Although the amount of proliferation can directly impact the overall size of the eye, the distortion in shape and proportions occurring in hyperopia and myopia must also be considered. Computational modelling suggests that CMZ-mediated growth can cause spherical distortion unless properly regulated [48]. Differential and controlled cell division along the radial axis (along the peripheral to central retinal axis) not only spatially separates one daughter cell in and out of the niche to regulate and maintain constant RSCs/RPCs numbers [42] but also results in eye shape maintenance as new cells are added [48]. How this division axis is regulated in RSC/RPCs is unknown, but future research could evaluate regulators of mitotic spindle orientation in the CMZ [132]. If external pressures are applied, in the case of sclera rearrangement, cell division axes, however, may be less important as organ geometry dictates the directional growth [48]. These aspects of CMZ proliferation should be considered when modelling the possible mechanisms leading to eye shape changes in visual disorders. 

## 8. The CMZ and Its Potential to Prevent Retinal Disease 

If RSCs are present and active in the mature retina, are they capable of mobilizing to combat retinal disease? As seen in other tissues, intrinsic stem cells with homeostatic properties are often able to switch functional states to promote regeneration [37]. One might hypothesize that teleost fish and amphibians harbouring an active CMZ niche would have a significant capacity to mitigate against retinal disease. However, the evidence supporting this hypothesis remains inconclusive. This is because, despite the ability of zebrafish to continually produce new retinal neurons at the margin and even regenerate central neurons through Müller glia activation [10,133], the zebrafish retina is still vulnerable to retinal pathologies. In fact, many retinal degenerative conditions have been faithfully modelled using zebrafish [134,135], and retinal injury does not affect the proliferation rate or mode of division (i.e., asymmetric division) of RSCs in the CMZ [41], suggesting that the CMZ is mostly unresponsive to these types of injury cues. In regeneration-capable species, including teleost fish and amphibians, the CMZ does not appear to be the primary source of RSCs that are induced to respond to retinal injury. *Xenopus laevis*, for example, rely on the transdifferentiation of RPE cells into NR cells to regenerate lost retinal tissue [8,62]. In zebrafish, Müller glia dedifferentiate, re-enter the cell cycle and proliferate to regenerate retinal cells in response to injury [6,136]. Recently, *Xenopus laevis* also showed regeneration capabilities from Müller glia after a needle poke retinal injury or genetic ablation of photoreceptors [137]. While similar transcription factors and signalling pathways are commonly involved in regulating CMZ RSCs and activated Müller glial [6,10,87], the reason why CMZ cells are not mobilized in a regenerative response remains unknown. Interestingly, however, studies in *Xenopus tropicalis* show regeneration of the retina can occur through RSC/RPCs in the CMZ but only in relatively extreme circumstances of a near-complete extirpation of the retina [138]. 

These diverse modes of retinal repair in some species may correlate primarily with the type of injury. Most injury paradigms assessing proliferation use mechanical, light or pharmacological damage or selective ablation to induce retinal injury [134]. However, very few studies have examined the activation or proliferative changes occurring in genetic degenerative models of similar human diseases. In one zebrafish study modelling Bardet-Biedl syndrome (BBS), a condition resulting in retinal degeneration due to defects in the photoreceptor outer segments, it was shown that little regenerative response was initiated from Müller cells following cone photoreceptor loss, despite displaying inflammatory responses [139]. Furthermore, high-intensity light damage could stimulate Müller glia-mediated photoreceptor regeneration in these mutants but only to pre-light injury levels [139]. A different study, using a cone and rod degenerative model, showed that Müller glial cells exhibited reactive gliosis before eventually transitioning into a regenerative state a few weeks following photoreceptor cell death due to activation by TNFα signalling [140]. Although these experiments primarily focused on regeneration from Müller glia, the same questions can be considered for the CMZ to examine how its activity can be altered under injury or disease conditions and specifically whether its proliferative kinetics are changed to match the level of degeneration. Another point to consider about possible CMZ-mediated repair is that the CMZ usually produces a cohort of new retinal cells distributed in a columnar fashion throughout all three layers [46]. Unlike Müller glial-dependent regeneration, where specifically damaged cell types (e.g., photoreceptors) are specifically replaced, CMZ cells may not be adapted to behave in such a manner. Biased production of particular cell types has not been previously shown from the CMZ, although, as mentioned above, it is known that Notch–*Atoh7* signalling can influence the cell fate of CMZ cells [110]. While future application of this knowledge may be used, for example, to bias CMZ production of cells to photoreceptors in models of rod-cone dystrophies, major limitations remain before we can consider how to truly capitalize on the therapeutic potential of marginal cells of the human retina. As a first step, we should consider how CMZ cells can be further activated in degeneration zebrafish models to mitigate the effects of retinal cell loss and whether this can be effective to slow down disease progression. 

## 9. Conclusions and Future Perspectives

Despite numerous fundamental insights on the CMZ and CMZ-like tissues, why different species exhibit different in vivo RSC behaviour is still not well understood. There are hints at differences in proliferative potential and multipotency, either due to differences in gene expression or signalling mechanisms in the retina, but this is not clear as comparative studies that manipulate genes or signalling pathways in different species, namely outside teleost fish, are limited. As described, the above research over the years has greatly improved our understanding of what controls proliferative dynamics in species with an active CMZ, mainly from teleost fish and amphibians, but the translation of the research found in zebrafish/amphibian models to other animal models, including mice and vice versa is lacking. For example, more studies examining how overexpression of genes, signalling molecules or a combination of factors in non-active CMZ could transform a CMZ or ciliary margin region’s activity should be considered. Application of drugs may be particularly desirable given their ease of application in humans. Furthermore, mammalian species, such as mice, appear to have a CMZ-like region at younger stages that are largely lost into adulthood. Future studies should consider whether manipulations to factors in the CMZ early in retinal development are required to maintain the CMZ population for longer before it is lost and to determine the impact of this on normal retinal development and maintenance. Another question to ask is whether the CB cell type could be stimulated enough to sufficiently produce a source of continuous RSC supply. Going forward, the field will also greatly benefit by taking a cross-species comparative approach, especially considering the development of tools such as CRISPR, which have the potential to be applied to unconventional animal models [141]. We would argue that the comparative approach—exemplified in the papers highlighted in this review—offers the best opportunity to learn from the natural phenotypic variation in species with unique evolutionary adaptations for retinal tissue maintenance through CMZ or CMZ-like tissues in order to advance the frontier of human retinal repair research. Recent studies examining the CMZ in unconventional species, including reptiles, have started to frame new questions on the conservation or lack thereof of the CMZ or CMZ-like niche and could inform on commonalities required for an active CMZ. Moreover, with the advancement of single-cell RNA sequencing [142,143] and spatial transcriptomics [144], it is now possible to obtain the transcriptome of cells found at the periphery of the retina in multiple different species and search for similarities in their gene expression profiles. Not only could this potentially identify with greater accuracy cells that exhibit similar stem cell characteristics across species and their location in the retina, but it may also identify novel mechanisms through which these cells are differentially regulated. For example, single-cell RNA sequencing on ciliary margin or CMZ cells from mammalian eyes compared to teleost eyes throughout different developmental timepoints could unbiasedly show which gene pathways show the greatest differences in activities. Using this information, genetic manipulation or designed drug targets against these pathways may provide novel therapeutics to stimulate RSCs in vivo. Furthermore, these future studies may identify how such mechanisms contribute to disease phenotypes when altered, such as high-grade myopia or hyperopia and how they could be avoided, or how they could be utilized to promote the proliferation in species that do not normally show regenerative capabilities. Although RSCs seem to have different ‘retirement plans’ between species, future research is poised to uncovering the detailed ‘investment portfolios’ of each one.

**Table 1:** Known genes and signalling pathways linked to CMZ/RPC regulation that also result in dystrophic eye growth conditions, myopia or hyperopia.

Myopia		
**Gene**	**Link to Myopia/Hyperopia**	**Association with the CMZ or RPC Activity**
Signalling Molecule		
Shh	Treatment with molecule increases axial length [131]	Target molecules are expressed in the CMZ [44,95] Can increase RPC proliferation in the CMZ [96,100]
Glucagon	Antagonist against molecule results in myopia-like changes [107,108]	Inhibits CMZ proliferation; antagonists promote proliferation [107,108,109]
IGF-1	Increased in form-deprivation myopia [129] Treatment with molecule causes extreme myopia [91]	Receptor is expressed in the CMZ [92,93,94] Manipulation of the ligand or receptor promotes proliferation of RPCs at the CMZ [94]
Wnt	Increased in form-deprivation myopia [129,130]	Expressed in the CMZ; Known to inhibit CMZ proliferation [95,101,102,103,145]
Mutated Gene		
*Lrp2*	Mutation causes myopia in zebrafish [122]	Depletion of CMZ cells over time [122]
*ZNF644*	Exome Sequencing links *ZNF644* mutations in humans to high-grade myopia [120] Mice mutants exhibit myopia [119]	Loss of *znf644* in zebrafish results in prolonged proliferation of embryonic RPCs [74]
**Hyperopia**		
Signalling Molecule		
Shh	Inhibition of molecule decreases axial length [131]	Can decrease RPC proliferation by promotion of cell cycle exit [98]
Wnt	*MRFP* is a frizzled-related protein (receptor of Wnt) mutated in hyperopia	Known to inhibit CMZ proliferation [95,101,102,103,145]
Mutated Gene		
*MRFP*	Sequencing indicates gene is mutated in hyperopic patients [123,126]	Expressed in the CE at the margin in zebrafish [124]
*Prss56*	Sequencing indicates hyperopic patients have mutations in *Prss56* [123,127]	*Prss56* is expressed in RPCs in the developing retina [128]

## Abbreviations

CMZ	Ciliary/circumferential marginal zone
NR	Neural retina
RPE	Retinal pigmented epithelium
CE	Ciliary epithelium
CB	Ciliary body
RPC	Retinal progenitor cells
RSCs	Retinal stem cells
PCNA	Proliferating cell nuclear antigen
RCJ	Retinociliary junction
SVZ	Subventricular zone
SGZ	Subgranular zone
ArCoSs	Arched continuous stripes
INL	Inner nuclear layer
ONL	Outer nuclear layer
HDACi	Histone deacetylase inhibitors
BDNF	brain derived neurotropic factor
Dnmt1	DNA methyltransferase 1
IGF-1	Insulin-like growth factor-1
Igf1r	IGF1 receptor
Shh	Sonic hedgehog
Ptch2	Patched2
Rx/rax	Retina and Anterior Neural Fold Homeobox
Chx10/Vsx2	Ceh-10 Homeodomain Containing Homolog/Visual System Homeobox 2
Pax6	Paired Box 6
Sox2	(Sex determining region-Y)-box 2
pH3	phospho-histone H3
Sox9	SRY-Box Transcription Factor 9
Msx1	Msh Homeobox 1
Zic2	Zic Family Member 2
CRALBP	Cellular retinaldehyde- binding protein
GFAP	Glial fibrillary acidic protein
dmbx1a	Diencephalon/mesencephalon homeobox 1a
Ezh2	Enhancer Of Zeste 2
PRC2	Polycomb repressive complex 2
Gli1/3	GLI Family Zinc Finger 1/3
Her9	Hairy-related 9
Six3/6	SIX homeobox 3/6
GLP1	Glucagon-like peptide-1
Znf644	Zinc finger protein 644
TNFα	Tumor necrosis factor alpha
BrdU	Bromodeoxyuridine
EdU	Ethynyl Deoxyuridine
